# The Relationships Between Diet Quality Index, Appendicular Skeletal Muscle Mass, and Metabolic Syndrome in a Population with Metabolic Dysfunction-Associated Steatotic Liver Disease: A Single Center from the Yucatan Peninsula

**DOI:** 10.3390/metabo16080546

**Published:** 2026-08-01

**Authors:** Roberto Lugo, Ana Ligia Gutiérrez-Solis, Paul Góngora-Chan, Ricardo Emmanuel Jimeno-Figueroa, Brenda Pacheco-Hernández, Rodolfo Chim-Aké, Mayra Vera-Aviles, Dayana Williams-Jacquez, Marlene Chaurand-Lara, Jorge Arturo Valdivieso-Jimenez, Isabel Medina-Vera, Martha Guevara-Cruz, Azalia Avila-Nava

**Affiliations:** 1 Unidad de Investigación, Hospital Regional de Alta Especialidad de la Península de Yucatán, Servicios de Salud del Instituto Mexicano del Seguro Social para el Bienestar (IMSS-BIENESTAR), Mérida 97130, Yucatán, Mexico; jlugo.hraepy@imssbienestar.gob.mx (R.L.); agutierrez.hraepy@imssbienestar.gob.mx (A.L.G.-S.); brenda.pachecoherndez@gmail.com (B.P.-H.); rodolfochim@hotmail.com (R.C.-A.); williamsdayana@gmail.com (D.W.-J.); dra.chaurand@gmail.com (M.C.-L.); 2 Departamento de Radiología, Hospital Regional de Alta Especialidad de la Península de Yucatán, Servicios de Salud del Instituto Mexicano del Seguro Social para el Bienestar (IMSS-BIENESTAR), Mérida 97130, Yucatán, Mexico; medgeneral.drpaul@hotmail.com (P.G.-C.); jimeno.fig@gmail.com (R.E.J.-F.); 3 Department of Physiology, Anatomy and Genetics, University of Oxford, Sherrington Building, Parks Road, Oxford OX1 3PT, UK; mayra.veraaviles@dpag.ox.ac.uk; 4 Servicio de Medicina Interna, Hospital Regional de Alta Especialidad de la Península de Yucatán, Servicios de Salud del Instituto Mexicano del Seguro Social para el Bienestar (IMSS-BIENESTAR), Mérida 97130, Yucatán, Mexico; dr.arturo.valdivieso@gmail.com; 5 Departamento de Endocrinología, Hospital Regional Dr. Mario Cárdenas de la Vega, Instituto de Seguridad y Servicios Sociales de los Trabajadores del Estado (ISSSTE), Culiacán 80230, Sinaloa, Mexico; 6 Departamento Metodología de Investigación, Instituto Nacional de Pediatría, Ciudad de Mexico 04530, Mexico; imedinav@pediatria.gob.mx; 7 Departamento de Fisiología de la Nutrición, Instituto Nacional de Ciencias Médicas y Nutrición Salvador Zubirán, Ciudad de México 14080, Mexico; martha.guevarac@incmnsz.mx

**Keywords:** diet quality, muscle mass, metabolic alterations

## Abstract

**Background/Objectives:** Metabolic dysfunction-associated steatotic liver disease (MASLD) is an increasingly prevalent disease closely linked to dietary habits and body composition, particularly reflected in lean mass and markers such as appendicular skeletal muscle mass (ASM). Low ASM can subsequently trigger metabolic abnormalities such as metabolic syndrome (MetS). This study aimed to evaluate the relationships between the diet quality index, ASM, and MetS in individuals with MASLD from the Yucatan Peninsula. **Methods:** A cross-sectional study was conducted in subjects with a MASLD diagnosis. Diet quality index, anthropometric and biochemical parameters were evaluated. Body composition analysis was utilized to estimate ASM. Correlations between ASM and anthropometric/biochemical parameters were analyzed. Furthermore, comparative analyses were performed between groups stratified by the presence of MetS and low ASM. **Results**: Fifty subjects were included in the study. Participants exhibited a significantly low diet quality index (57.6 ± 9.78 vs. 100, *p* < 0.001), alongside a high prevalence of both low ASM and MetS. ASM was positively correlated with BW, WC, and protein mass (*p* < 0.001), as well as with creatinine and ALT (*p* < 0.05), and negatively correlated with body fat percentage and TG (*p* < 0.05). Subjects with low ASM showed significant differences in BW, waist circumference, protein mass, and energy intake compared to those with normal ASM (*p* < 0.05). Individuals with MetS had higher glucose and blood pressure compared to non-MetS (*p* < 0.05). Additionally, among patients with MASLD and MetS, those with low ASM exhibited higher glucose and systolic blood pressure compared to those with normal ASM (*p* < 0.05). **Conclusions:** Our study demonstrates that adults with MASLD from the Yucatan Peninsula exhibited suboptimal diet quality according to the DQI-I, with a high prevalence of low ASM and MetS. These findings highlight the potential role of the muscle–liver axis in metabolic dysfunction in MASLD.

## 1. Introduction

Contemporary lifestyle modifications have been increasingly linked to the development of cardiometabolic diseases. Prominent among these risk factors are low diet quality, altered body composition, and sedentary behavior [1].

A balanced diet plays a fundamental role in regulating essential metabolic processes. In contrast, a poor-quality diet is characterized by excessive consumption of ultra-processed foods, saturated fats, and carbohydrates (CHO). In this sense, the Diet Quality Index-International (DQI-I) has emerged as a useful tool for evaluating dietary patterns and their relationships with metabolic health and body composition [2].

Dietary modifications have been associated with differences in body composition, particularly regarding muscle mass depletion and body fat accumulation. Low muscle mass adversely affects metabolic regulation, insulin-stimulated glucose uptake, and lipid metabolism and contributes to the development of cardiometabolic disease [3]. In this context, appendicular skeletal muscle mass (ASM) reflects the lean soft tissue mass of the limbs, and ASM adjusted for body weight (BW), height squared or body mass index (BMI) has emerged as a valuable tool for quantifying skeletal muscle mass as a marker of metabolic health [4].

Additionally, during excessive body fat accumulation, white adipose tissue can undergo functional changes and lose its ability to store lipids, increasing lipolysis in adipose tissue and resulting in elevated levels of circulating free fatty acids, leading to a process known as lipotoxicity [5]. This process has been linked to the development of steatotic liver. Excessive accumulation of lipids in hepatocytes disrupts many metabolic pathways and affects lipid and carbohydrate metabolism [6].

This interconnected cluster of abnormalities has been defined as metabolic dysfunction-associated steatotic liver disease (MASLD), previously termed non-alcoholic fatty liver disease (NAFLD). MASLD is among the most frequent causes of chronic liver diseases in low- and lower-middle-income countries [7]. It is defined as steatotic liver disease characterized by the presence of one or more cardiometabolic risk factors and the absence of harmful alcohol intake. Among the cardiometabolic risk factors are central obesity (waist circumference (WC) >90 cm in men and >80 cm in women), high levels of triglycerides (TG) (>150 mg/dL), low levels of high-density lipoprotein cholesterol (HDL-C) (<39 mg/dL in men and <50 mg/dL in women) and elevated blood pressure (BP) (>130/85 mmHg) [8].

These alterations are also used to diagnose another pathological condition, metabolic syndrome (MetS). Several studies have reported a high prevalence of MetS among Mexican adults, specifically in the Yucatan Peninsula population [9,10]. Overall, the clinical implications of a low-quality diet and physical inactivity extend beyond adverse body composition and muscle mass reduction; these factors disrupt fundamental metabolic pathways and profoundly impact the incidence and mortality rates of cardiovascular disease and type 2 diabetes (T2D), the leading causes of death worldwide [11]. Therefore, the aim of the present study was to determine the relationships among DQI-I, ASM, and MetS in a population with MASLD from the Yucatan Peninsula.

## 2. Materials and Methods

### 2.1. Study Design and Population

This cross-sectional study with prospective enrollment was conducted at the Hospital Regional de Alta Especialidad de la Península de Yucatán, IMSS-Bienestar, between June 2024 and June 2025. The study was designed to identify associations among diet quality, appendicular skeletal muscle mass, and metabolic syndrome. Individuals with a confirmed diagnosis of hepatic steatosis based on ultrasonography were eligible for inclusion. The exclusion criteria included current chemotherapy and/or radiotherapy, the use of more than three medications associated with drug-induced liver injury, and a history of alcoholism or smoking. Additionally, individuals with human immunodeficiency virus infection, hepatitis B or C infection, cirrhosis, or pregnancy were excluded.

### 2.2. Sample Size

The sample size was performed using G*Power version 3.1.9.6 based on a moderate-to-large effect size, aiming for a statistical power of 80% and a significance level of 0.05. A minimum of 46 participants were needed.

### 2.3. Sociodemographic and Clinical Characteristics of the Population

Sociodemographic data, including age, sex, marital status, area of residence, and educational level, were collected. Lifestyle characteristics such as alcohol consumption, smoking status, and physical activity were also recorded. Clinical conditions associated with MASLD, including T2D, hypertension (HT), urolithiasis (UL), glomerular filtration rate (GFR), and systolic blood pressure (SBP) and diastolic blood pressure (DBP), were obtained from all participants.

### 2.4. Anthropometric Characteristics and Body Composition

Body weight (BW), height, and waist circumference (WC) were measured following standardized procedures [12]. Body mass index (BMI) was calculated as weight in kilograms divided by height in meters squared (kg/m^2^).

Body composition parameters, including body fat percentage, fat mass, and lean mass and appendicular skeletal muscle mass (ASM), were assessed using a multifrequency bioelectrical impedance analyzer (InBody 120, Seoul, Republic of Korea). To evaluate body composition, participants were instructed to avoid vigorous physical activity for at least 12 h, alcohol consumption during the previous 24 h, and excessive fluid intake. They were also instructed to empty their bladder immediately before the assessment.

All body composition assessments were performed in the morning under standardized conditions. Participants fasted for 10 h and were evaluated barefoot while wearing light clothing. Measurements were performed with participants standing on the device’s electrodes and maintaining an upright position, according to the manufacturer’s instructions. All measurements were conducted by a trained nutritionist.

### 2.5. Serum Biochemical Parameters

Blood samples were collected after a 10 h fast. The samples were centrifuged at 1200× *g* for 10 min to separate the serum and subsequently stored at −80 °C until analysis. Glucose, creatinine, total cholesterol (TC), TG, HDL-C, low-density lipoprotein cholesterol (LDL-C), total lipid (TL), and transaminase levels were measured using colorimetric assays with a Cobas c111 analyzer (ROCHE^®^ Cobas c111, Rotkreuz, Switzerland). The glomerular filtration rate was estimated using the CKD-EPI formula [13].

### 2.6. Diet Quality Index-International

Dietary intake was assessed using 24 h dietary recall to calculate a diet quality index. The index evaluates four major components of the diet quality, variety, adequacy, moderation, and overall balance, with total scores ranging from 0 to 100 points [2]. The variety component (a maximum of 20 points) included food group diversity based on the serving of meat, cereals, fruits, vegetables, milk, and legumes. The adequacy component (maximum of 40 points) assesses the intake of vegetables, fruits, grain, fibers, proteins, iron, calcium, and vitamin C, expressed in kilocalories per day. The moderation component (maximum of 30 points) evaluated total fat, cholesterol, sodium, saturated fat, and the consumption of foods with empty calories. Finally, the overall balance component (maximum of 10 points) considers the proportionality of macronutrients (proteins, lipids, and CHO) and fatty acid composition.

### 2.7. Appendicular Skeletal Muscle Mass

ASM was calculated as the sum of the lean soft tissue mass of both arms and both legs. Low ASM was defined using cut-off points of <20 kg for men and <15 kg for women, according to the European Working Group on Sarcopenia in Older People (EWGSOP2, 2019). Additionally, the ASM adjusted for height squared (ASM/height^2^) was calculated, with cut-off points of <7.0 kg/m^2^ for men and <5.5 kg/m^2^ for women [14].

### 2.8. Metabolic Syndrome

Participants were classified into those with or without MetS, according to the International Diabetes Federation [15]. MetS was defined as the presence of central obesity (WC ≥90 cm in men and ≥80 cm in women) plus at least two of the following criteria: TG ≥ 150 mg/dL, reduced HDL-C (<40 mg/dL in men and <50 mg/dL in women), elevated BP (SBP ≥130 mmHg or DBP ≥ 85 mmHg, or treatment for previously diagnosed HT), and elevated fasting plasma glucose (≥100 mg/dL or previously diagnosed T2D).

### 2.9. Statistical Analysis

The normality of continuous variables was assessed using the Shapiro–Wilk test (Supplementary Table S1). Data are presented as the mean ± standard deviation or median and interquartile range (Q1–Q3] based on distribution. Comparisons with reference values were performed by one-sample t tests, and comparisons between groups were performed using the Mann–Whitney U test. Differences according to MetS status and ASM categories (low vs. normal) were evaluated by the Kruskal-Wallis test with post hoc Dunn’s test. Spearman’s rank correlation coefficient was used to assess the associations between serum biochemical, anthropometric, and body composition parameters and ASM and ASM/height^2^. A *p*-value < 0.05 was considered to indicate statistical significance.

## 3. Results

**Figure 1:**
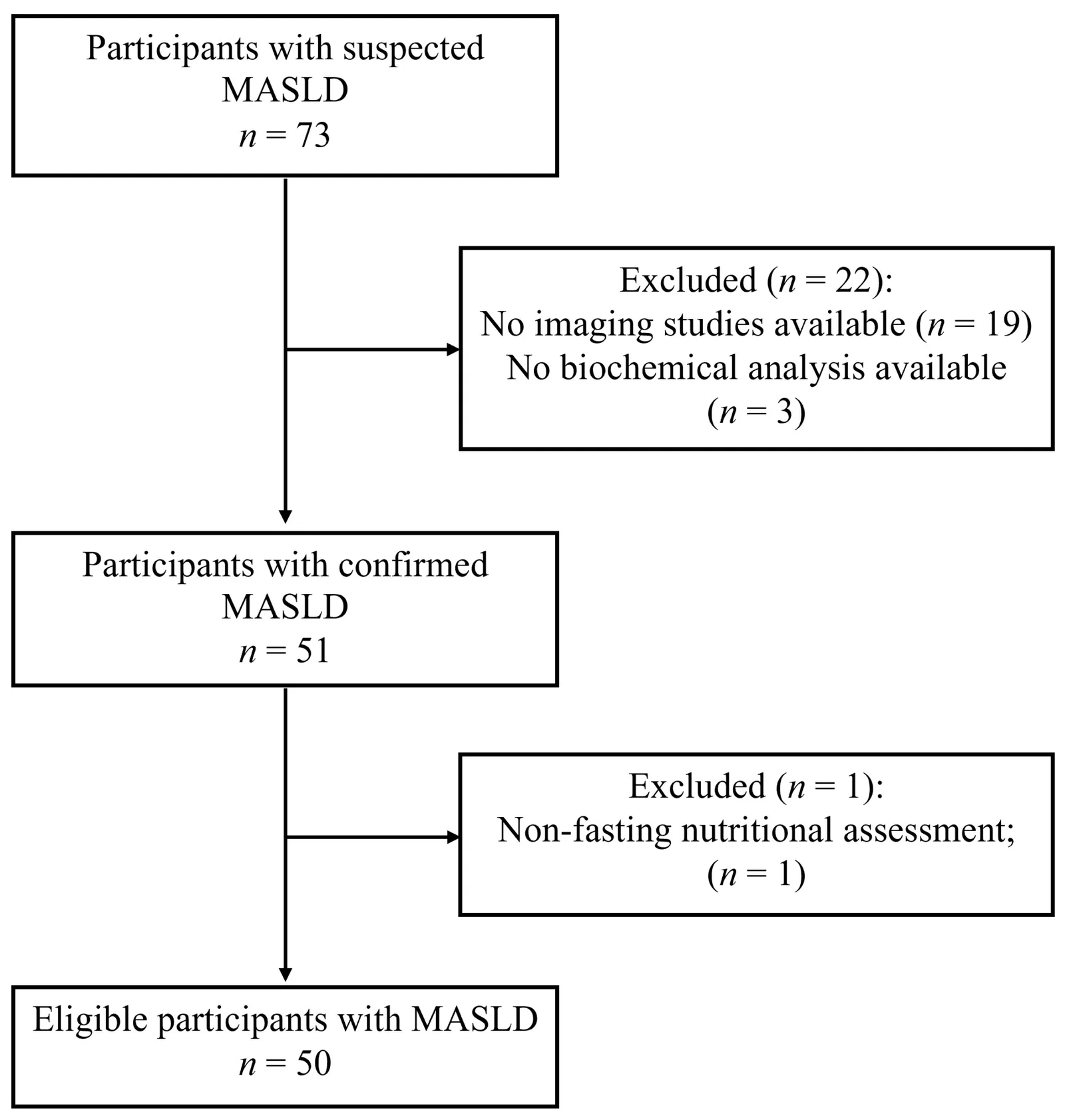
Flow diagram of participant selection.

A total of 50 individuals with confirmed MASLD were included in the study, with a mean age of 41.8 ± 11.8 years; 58% (*n* = 29) were female (Figure 1).

All participants were native residents of the Yucatan Peninsula, and the majority were residents of urban areas (52%). MASLD diagnosis was confirmed through imaging studies, clinical evaluation, and serum biochemical analysis. With respect to lifestyle characteristics, 20% of participants reported occasional alcohol consumption, although their intake did not exceed the recommended daily limits (30 g/day for men and 20 g/day for women) [16]. In addition, 26% of the participants reported engaging in regular physical activity. Reduced GFR (<90 mL/min/1.73 m^2^) and UL were the most frequent alterations associated with MASLD in this population. Serum biochemical analysis also revealed increased TG, LDL-C, and TL levels. The general characteristics of the study population are summarized in Table 1.

**Figure 2:**
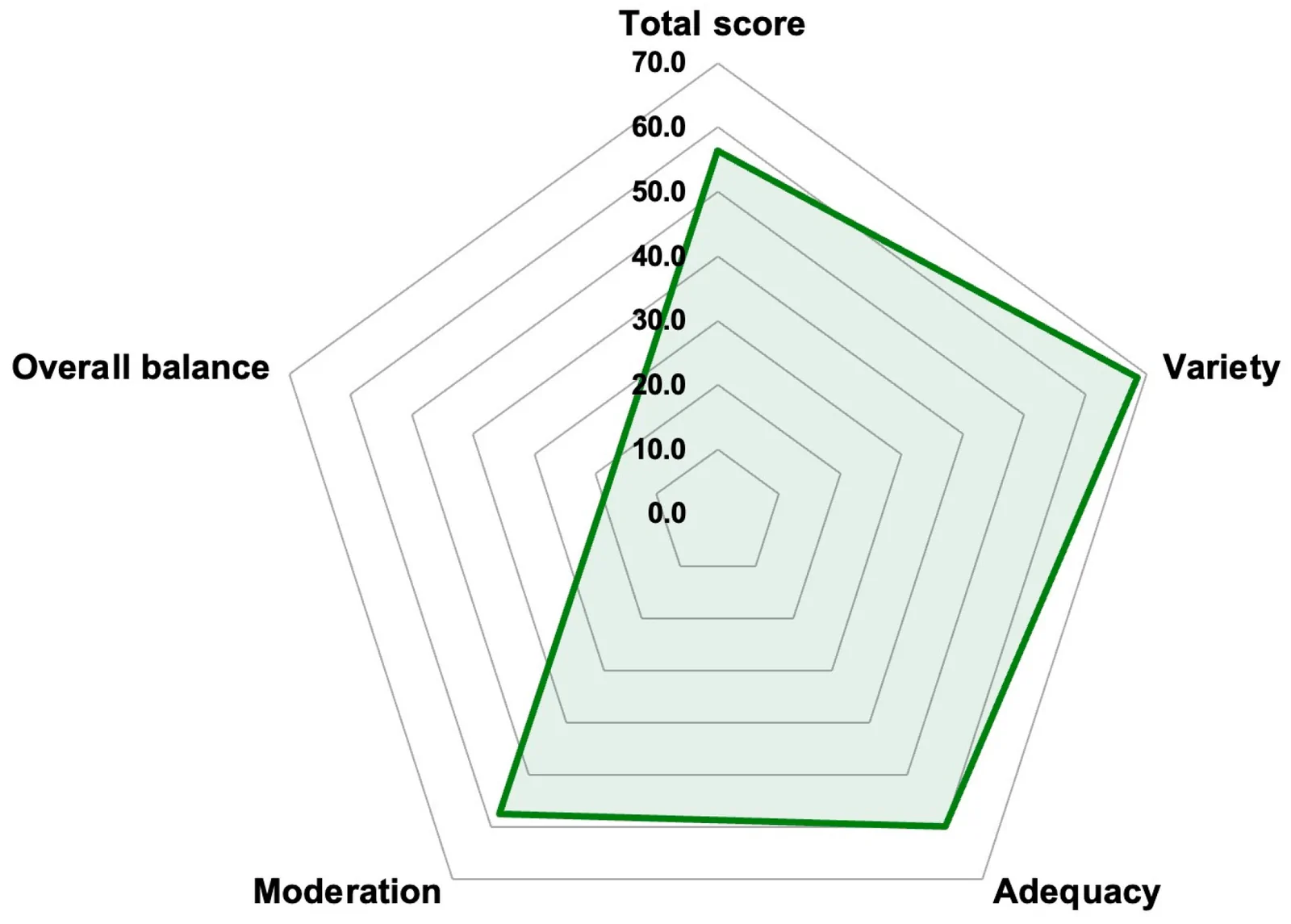
Radar plot of Diet Quality Index-International component scores in population with MASLD.

The mean total DQI-I score was lower than the maximum reference value (57.6 ± 9.78 vs. 100, *p* < 0.001), indicating suboptimal overall diet quality in the study population. As shown in Figure 2, the variety component reached values close to the maximum reference score, suggesting adequate dietary diversity, although not necessarily healthy food choices. In contrast, the overall balance component had the lowest score among all DQI-I domains, reflecting an inadequate distribution of macronutrients (proteins, lipids, and CHO), unfavorable fatty acid proportions, and a metabolically unbalanced dietary pattern.

Compared with the published reference values reported by Kim et al. (2003) [2], participants in the study showed significantly lower scores for the DQI-I domains for variety and adequacy but higher scores for moderation (all *p* < 0.001) (Table 2).

**Figure 3:**
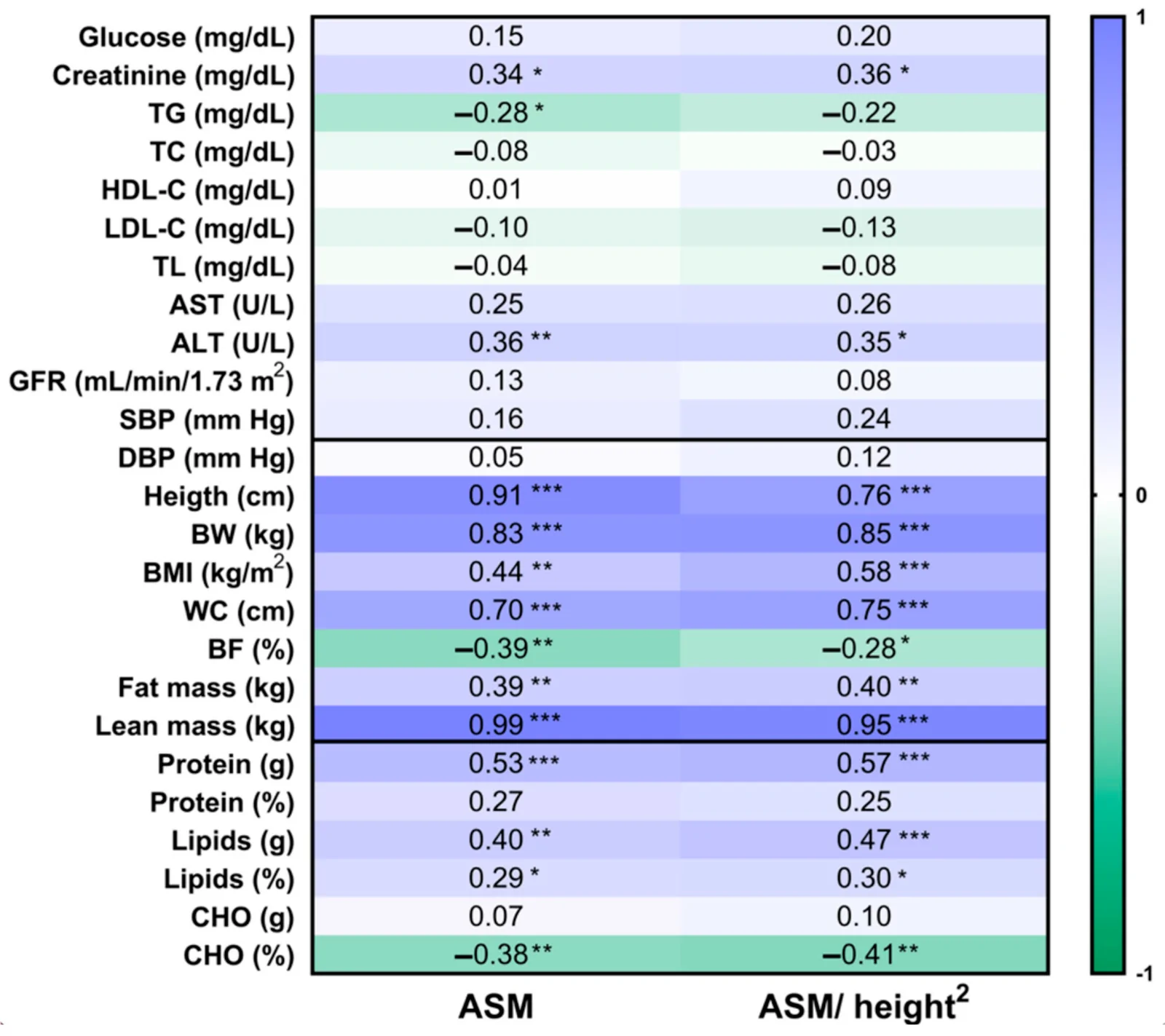
Correlations of ASM and ASM/height^2^ with biochemical parameters, body composition, and dietary intake in population with MASLD. Statistically significant are represented as follow: *: *p* < 0.05; **: *p* < 0.01; ***: *p* < 0.001. ASM: Appendicular skeletal muscle mass; ASM/height^2^: ASM adjusted for height squared; MASLD: Metabolic dysfunction-associated steatotic liver disease; TG: Triglycerides; TC: Total cholesterol; HDL-C: High-density lipoprotein cholesterol; LDL-C: Low-density lipoprotein cholesterol; TL: total lipids; AST: Aspartate aminotransferase; ALT: Alanine aminotransferase; GFR: Glomerular rate ratio; SBP: Systolic blood pressure; DBP: Diastolic blood pressure; BW: Body weight; BMI: Body mass index; WC: Waist circumference; CHO: Carbohydrates.

Given that diet plays a significant role in body composition, particularly muscle mass, we performed correlation analyses between ASM and biochemical, anthropometric, body composition, and dietary intake variables. The results reveal strong positive correlations between ASM and height, BW, WC, and lean mass (*p* < 0.001). Moderate positive correlations were observed between ASM and protein intake (g) (*p* < 0.001), BMI, and lipid intake (g) (*p* < 0.01), whereas weak positive correlations were identified with ALT (*p* < 0.01) and creatinine (*p* < 0.05) (Figure 3). Conversely, ASM was negatively correlated with body fat percentage, CHO intake (%) (*p* < 0.01), and TG levels (*p* < 0.05). Interestingly, after adjusting ASM for height^2^, only 4% of participants were classified as having low muscle mass, indicating that most subjects maintained relative muscle mass despite MASLD (Supplementary Table S2). Similar correlation patterns were observed when the ASM/height^2^ was analyzed.

Participants were subsequently categorized into low- and normal-ASM groups to evaluate whether reduced muscle mass was associated with an unfavorable metabolic or dietary profile. Thirty-three percent were classified as having low ASM, of whom 82.3% (*n* = 14) were women, while 45.4% (*n* = 15) had normal ASM. Compared with those with normal ASM, subjects who were older in age and had higher LDL-C levels were found in the low-ASM group, whereas lower AST and ALT levels, lean mass, protein intake, height, BW, WC, BMI, fat mass, and lipid intake were observed in the low-ASM group (Table 3).

Sex-related differences in age and WC were evaluated among individuals with low and normal ASM. With respect to age, significant differences were observed among women with low and normal ASM (48.5 [40.2–54.0] vs. 33.0 [29.0–45.0] years; *p* = 0.040), and a similar trend was observed among men (59.0 [40.0–61.0] vs. 35.0 [31.0–45.5] years; *p* = 0.059). For WC, significant differences between the low- and normal-ASM groups were observed for both men (99.0 [87.0–102.0] vs. 108.0 [102.0–124.0] cm; *p* = 0.044) and women (92.2 [86.2–97.3] vs. 99.0 [92.1–107.0] cm; *p* = 0.017).

Based on these findings, additional sex-stratified analyses were performed for participants with normal ASM. Significant differences were observed in BW between men and women (98.0 [89.9–115.0] vs. 90.0 [81.2–93.1] kg; *p* = 0.019). However, no significant differences were found in BMI (35.2 [32.5–38.8] vs. 35.1 [33.2–36.4] kg/m^2^; *p* = 0.998), fat mass (39.0 [34.6–46.6] vs. 44.8 [38.1–50.8] kg; *p* = 0.691), or lipid-intake percentage (29.0 [25.7–34.3] vs. 34.3 [27.0–37.0]; *p* = 0.562).

In this cohort, 58% (*n* = 29) of the participants met the diagnostic criteria for MetS; of these, 59% (*n* = 17) were men, and 41% (*n* = 12) were women. Baseline clinical, biochemical, anthropometric, and dietary characteristics according to MetS status are presented in Table 4. Compared with participants without MetS, those with MetS had significantly higher fasting glucose concentrations, SBP, DBP, and CHO intake.

To further explore the combined relationship between metabolic syndrome and muscle mass, participants were stratified into four groups according to MetS status and ASM classification (Table 5). Significant differences among the groups were observed for TG levels, ALT levels, DBP levels, several anthropometric variables, and dietary intake. With respect to metabolic parameters, TG concentrations were significantly lower in participants without MetS and with normal ASM than in the remaining three groups. ALT concentrations differed mainly because participants with MetS and low ASM presented the lowest values, whereas the ALT concentrations of the other three groups were comparable. In addition, DBP was significantly higher among participants with MetS regardless of ASM classification.

To evaluate the discriminatory performance of ASM/height^2^ for discriminating participants with and without MetS, an exploratory ROC analysis was performed using MetS as the reference outcome. The optimal exploratory threshold and its corresponding diagnostic performance are presented in Supplementary Table S3 and Figure S1. Given the exploratory nature of this analysis and the limited sample size, these findings should be interpreted as hypothesis-generating and require validation in larger cohorts.

Anthropometric differences were predominantly associated with ASM classification. Regardless of MetS status, compared with participants with low ASM, participants with normal ASM consistently presented greater height, BW, BMI, WC, fat mass, lean mass, and protein intake (g and %) and a higher percentage of dietary lipids. Conversely, participants with low ASM had a significantly greater proportion of CHO intake.

Finally, associations between DQI-I domains and ASM classification were evaluated. Compared with participants with normal ASM, participants with low ASM had significantly higher moderation scores and higher total DQI-I scores (*p* < 0.05). No significant differences were observed in the variety or adequacy domains, although a trend toward higher overall balance scores was identified in the low-ASM group (Supplementary Table S4).

## 4. Discussion

The present study evaluated the relationships among diet quality, ASM, and MetS in adults with MASLD from the Yucatán Peninsula. The main findings demonstrate that this population exhibited poor diet quality according to the DQI-I, that one-third of participants presented low ASM, and that nearly half fulfilled the diagnostic criteria for MetS. Collectively, these findings highlight the close interaction between dietary patterns, body composition, and metabolic dysfunction in MASLD.

Body composition and metabolic abnormalities are associated with low consumption of fruits, vegetables, legumes, whole grains, and fiber-rich foods [17,18,19], as well as low physical activity [20]. Our results reveal that the mean DQI-I score observed (57.9 ± 9.78) was similar to that observed in Chinese adults with NAFLD (57.2 ± 11.5); this study also revealed that a decrease in 10 units in the DQI-I score increased the risk of developing NAFLD, even after adjusting for age and sex (OR: 1.24, 95% CI: 1.06–1.45; *p* = 0.009) [17]. These findings suggest that poor diet quality is a common feature among individuals with steatotic liver disease.

The analysis of individual DQI-I domains provided additional insights into the dietary habits of the study population. Variety, adequacy, moderation, and overall balance reached 68.5%, 61.0%, 58.7%, and 17.9% of their respective maximum possible scores. Compared with the published reference values reported by Kim S. et al. [2], participants in the study showed lower scores for the variety and adequacy domains, higher scores for moderation, and no significant differences in overall balance or the total DQI-I score. Dietary variety reflects a balanced diet, including a variety of protein sources and food groups, which are factors that may contribute to the prevention of metabolic disorders related to MASLD [17]. Our results reveal that the population with MASLD had a low-variety diet. This pattern is similar to that reported for the general Mexican population, which presented low adherence to the recommendations for the consumption of fruits, vegetables, and legumes and high consumption of sugary drinks, processed meats, and products high in saturated fats and/or added sugars, which have been linked to the development of conditions such as obesity, MetS, and MASLD [21].

In recent years, ASM has emerged as an important marker of skeletal muscle health because it provides a reliable estimate of total skeletal muscle mass and has been associated with insulin resistance, T2D, obesity, and increased cardiometabolic risk [22,23]. Within the context of MASLD, ASM is particularly relevant because this disease is currently regarded not only as a liver disorder but also as a manifestation of systemic metabolic dysfunction. Consequently, ASM may provide additional information regarding metabolic status, early stages of sarcopenic obesity, and cardiometabolic risk in subjects with MASLD [24,25].

Consistent with this concept, participants with low ASM had higher LDL-C concentrations and a greater proportion of dietary CHO intake than those with normal ASM. Previous evidence suggests that diets rich in refined CHO may increase hepatic lipogenesis and promote the production of atherogenic lipoproteins, while reduced muscle mass may impair glucose disposal, exacerbate insulin resistance, and contribute to metabolic dysfunction in MASLD [17,26]. Together, these findings support the concept that alterations in skeletal muscle mass and dietary quality coexist and may contribute to the metabolic abnormalities observed in patients with MASLD.

Age was another factor associated with ASM; subjects with low ASM were older than those with normal ASM. Age-related declines in skeletal muscle mass are well documented and have been attributed to physiological changes associated with aging, sex, ethnicity, reduced anabolic capacity, and progressive muscle loss [27,28]. Approximately one-third of the participants had low ASM despite belonging to a middle-aged population (mean age: 41.8 years), suggesting that alterations in muscle mass may already be present during early stages of adulthood in individuals with MASLD [29]. However, a study showed that ASM is associated with the severity of steatosis and significant fibrosis in MASLD in young adults <35 years [30]. In addition, sex-related differences in muscle aging have been previously reported [31,32]. Consistent with these observations, our sex-stratified analysis revealed significant age differences among women and a similar trend among men, suggesting that age may contribute to the observed differences in muscle mass among patients with MASLD. Although our findings support an association between ASM and metabolic alterations, the observed differences may also be partially influenced by age, as older participants naturally exhibit lower skeletal muscle mass and distinct metabolic characteristics.

Interestingly, participants with normal ASM did not necessarily exhibit a healthier metabolic profile. Compared with individuals with low ASM, subjects with normal ASM presented higher transaminase concentrations, BW, BMI, WC, fat mass, and dietary intake of proteins and lipids. The higher ALT and AST levels observed in this group may reflect greater hepatic fat accumulation or obesity-related low-grade inflammation rather than a direct effect of muscle mass itself. Banik et al. reported that chronic low-grade inflammation plays a central role in the pathogenesis of insulin resistance [33]. Furthermore, the anthropometric and body composition characteristics observed in participants with normal ASM suggest that preserved muscle mass frequently coexists with obesity and abdominal adiposity. These results are also reflected in the correlation analyses, which revealed positive correlations between ASM and ASM/height^2^ and the anthropometric variables but not with the percentage of body fat, for which a negative correlation was found (*ρ* = −0.28; *p* = 0.05). These results indicate that preserved muscle mass may coexist with increased adiposity and central fat accumulation in subjects with MASLD. Therefore, normal ASM should not necessarily be interpreted as a marker of metabolic health, highlighting the importance of evaluating muscle mass together with adiposity and dietary patterns when assessing metabolic dysfunction in MASLD [8].

Reduced muscle mass and impaired muscle metabolism increase systemic inflammatory signaling, consequently affecting skeletal muscle function, which may contribute to hepatic lipid accumulation, insulin resistance, and dysregulated TG metabolism [34]. Furthermore, lipid overload causes oxidative stress through excessive reactive oxygen species production and the induction of inflammatory processes, which contribute to impaired muscle quality and regeneration. In fact, a chronic low-grade inflammatory state activates the nuclear factor–κB signaling pathway and stimulates the activity of proteolytic systems such as the ubiquitin–proteasome and autophagy–lysosome pathways, accelerating muscle protein degradation [35].

Although the ASM represents the absolute amount of appendicular muscle tissue, an excess of adipose tissue may reflect an unfavorable metabolic environment characterized by impaired lipid metabolism and chronic inflammation, which contribute to impaired muscle maintenance and quality [35]. In this sense, regarding the relationship between ASM and MASLD, the studies come primarily from Asian populations. One of these studies revealed that lower ASM was associated with more severe hepatic steatosis and the presence of fibrosis [30]. Another population-based study in Korea revealed that lower ASM was strongly associated with the presence of MASLD in both men and women [36]. Similarly, in a cohort of Korean adults with NAFLD—the term used before MASLD—an accelerated loss of skeletal muscle mass was observed, which supports the existence of a bidirectional relationship between hepatic fat accumulation and progressive skeletal muscle deterioration [37]. Our results show that in the ASM adjusted for height squared, most participants were classified as having normal muscle mass. These findings may be partially explained by the anthropometric characteristics of the study population. However, it is important to consider that currently recommended ASM/height^2^ cut-off points have been developed in populations with different anthropometric, ethnic, and lifestyle characteristics from those of the Yucatan Peninsula [8]. In fact, some studies have identified specific cut-off points for our population, revealing differences among them [38,39]. Thus, these reference values may not fully capture low muscle mass in this population. Our findings suggest that population-specific reference values may be needed to improve the classification of low and normal ASM/height^2^ and to better characterize the relationship between muscle mass and metabolic dysfunction in Mexican adults. Overall, our findings indicate that preserved ASM does not necessarily represent a favorable metabolic phenotype in patients with MASLD. Instead, normal ASM frequently coexists with obesity, central adiposity, and metabolic abnormalities, highlighting the complex interactions among muscle mass, dietary habits, and metabolic dysfunction within the spectrum of MASLD.

MetS is recognized as one of the main pathophysiological drivers of MASLD. Indeed, the current definition of MASLD requires evidence of hepatic steatosis together with at least one cardiometabolic risk factor, including central obesity, T2D, hypertension, hypertriglyceridemia, or reduced HDL-C concentrations [40]. Consequently, MetS and MASLD frequently coexist and share common pathogenic mechanisms, such as insulin resistance, chronic low-grade inflammation, adipose tissue dysfunction, and altered lipid metabolism [41,42].

Epidemiological studies indicate that approximately 40–45% of individuals with MASLD meet the diagnostic criteria for MetS. Consistent with these reports, 46% of participants in the present study were classified as having MetS, a prevalence comparable to that reported in the Mexican adult population [43]. These findings reinforce the close relationship between metabolic dysfunction and hepatic steatosis in this population.

As expected, compared with those without MetS, participants with MetS had higher fasting glucose concentrations and elevated SBP and DBP [44]. Our findings support the concept that metabolic dysfunction is highly prevalent among patients with MASLD and further emphasize the close interrelationship between dietary habits, body composition, and liver health.

Hepatic metabolic dysfunction may promote muscle impairment through increased inflammation, altered nutrient availability, and metabolic stress, suggesting a bidirectional interaction between these organs [45]. Thus, when the population was stratified according to both MetS status and ASM classification, the most pronounced differences were observed in body composition and dietary intake rather than in the classical biochemical components of MetS. Compared with participants with low ASM, participants with normal ASM consistently presented greater BW, BMI, WC, fat mass, lean mass, and protein stores, regardless of MetS status. These findings suggest that ASM in this cohort was strongly influenced by overall body size and body composition. Interestingly, participants with MetS and normal ASM also had higher ALT concentrations than those with MetS and low ASM. Because ALT is closely associated with obesity, visceral adiposity, and hepatic steatosis, these results likely reflect a greater obesity-related hepatic burden independent of the effect of preserved muscle mass [46,47].

This study provides novel information regarding the relationships among diet quality, ASM, and MetS in subjects with MASLD from the Yucatan Peninsula. In addition, the simultaneous evaluation of dietary quality, biochemical parameters, body composition, and MetS status allowed for a comprehensive assessment of the interactions among nutritional, metabolic, and anthropometric factors. However, several limitations of this study should be acknowledged. First, the cross-sectional design allows for only the identification of associations between diet quality, ASM, and metabolic alterations and therefore precludes the establishment of temporal or causal relationships. Second, body composition was assessed using bioelectrical impedance analysis rather than dual-energy X-ray absorptiometry (DXA), which is considered the reference method for muscle mass assessment. Third, dietary intake was assessed using a single 24 h dietary recall. Although this method is widely accepted for assessing dietary intake in epidemiological studies, it may not fully reflect an individual’s habitual dietary intake because of day-to-day variability in food consumption. Furthermore, it is important to note that the sample size was calculated for the primary analysis of the overall study population rather than for the subgroup analyses. Consequently, these analyses should be considered exploratory, and their findings should be interpreted with caution. Finally, although the present study provides novel evidence regarding the relationships among diet quality, ASM, and metabolic syndrome in adults with MASLD, future studies with larger, multicenter cohorts are warranted to validate these findings, improve the precision of subgroup analyses, establish population-specific reference values for ASM/height^2^ in adults from the Yucatán Peninsula, evaluate alternative muscle mass indices such as ASM/BMI, and perform multivariable regression analyses to evaluate the independent contributions of diet quality, muscle mass, metabolic dysfunction, and lifestyle-related variables after adjustment for relevant confounding factors. Such studies will further strengthen the understanding of the complex interactions underlying MASLD in Latin American populations.

## 5. Conclusions

This study revealed that adults with MASLD from the Yucatan Peninsula exhibited suboptimal diet quality according to the DQI-I, with a high prevalence of low ASM and MetS. Our findings suggest an association between poor dietary quality, unfavorable body composition characteristics, and metabolic alterations in this population. Importantly, preserved ASM was not necessarily accompanied by a favorable metabolic profile, as individuals with adequate muscle mass frequently presented with increased adiposity, central obesity, and higher ALT concentrations. These findings highlight the clinical importance of evaluating muscle mass together with adiposity and metabolic biomarkers rather than considering muscle quantity alone as an indicator of metabolic health. Finally, longitudinal studies are needed to determine the temporal and causal relationships between dietary quality, changes in body composition, and metabolic dysfunction in individuals with MASLD.

## Figures and Tables

**Table 1:** General characteristics of the population.

Characteristics	Total *n* = 50
**Sociodemographic characteristics, *n* (%)**	
**Age** (years)	40 [31–51]
**Sex**	
Male	21 (42.0)
Female	29 (58.0)
**Marital status**	
Single	12 (24.0)
Married	24 (48.0)
Divorced	14 (28.0)
**Area of residence**	
Urban	26 (52.0)
Rural	24 (48.0)
**Education level**	
None	6 (12.0)
Primary/Secondary school	19 (38.0)
High school	9 (18.0)
College/University	16 (32.0)
**Lifestyle characteristics, *n* (%)**	
Alcohol consumption	10 (20.0)
Smoking status	2 (4.0)
Physical activity	13 (26.0)
**Clinical characteristics, *n* (%)**	
T2D	5 (10)
HT	8 (16.0)
UL	16 (32.0)
Reduced GFR	17 (34.0)
SBP (mm Hg)	122 [112–137]
DBP (mm Hg)	82.5 [76.0–90.5]
**Serum biochemical parameters**	
Glucose (mg/dL)	92.8 [84.0–103]
Creatinine (mg/dL)	0.80 [0.68–1.00]
TG (mg/dL)	156 [109–184]
TC (mg/dL)	187 [162–212]
HDL-C (mg/dL)	48.0 [40.1–55.5]
LDL-C (mg/dL)	110 [82.2–143]
TL (mg/dL)	259 [209–334]
AST (U/L)	21.7 [17.4–27.7]
ALT (U/L)	27.5 [16.5–34.7]
GFR (mL/min/1.73 m^2^)	106 [84.5–118]
**Anthropometrics and body composition**	
Height (cm)	156 [150–165]
BW (kg)	82.6 [71.5–93.0]
BMI (Kg/m^2^)	34.3 [30.2–36.9]
WC (cm)	101 [92.2–103]
Body fat (%)	45.5 [38.8–50.2]
Fat mass (Kg)	38.0 [30.7–45.2]
Lean mass (Kg)	25.4 [20.5–30.9]
**Daily intake**	
Proteins (g)	61.0 [50.0–84.9]
Proteins (%)	15.0 [13.0–20.0]
Lipids (g)	52.2 [36.4–66.6]
Lipids (%)	28.0 [24.0–34.0]
CHO (g)	228 [164–270]
CHO (%)	55 [46.0–64.0]
Energy intake (Kcal/day)	1594 [1272–1850]

Data are presented as the mean ± standard deviation or frequency and percentage or median [Q1–Q3]. T2D: Type 2 diabetes; HT: Hypertension; UL: Urolithiasis; GFR: Glomerular filtration rate; SBP: Systolic blood pressure; DBP: Diastolic blood pressure; TG: Triglycerides; TC: Total cholesterol; HDL-C: High-density lipoprotein cholesterol; LDL-C: Low-density lipoprotein cholesterol; TL: total lipids; AST: Aspartate aminotransferase; ALT: Alanine aminotransferase; BW: Body weight; BMI: Body mass index; WC: Waist circumference; CHO: Carbohydrates.

**Table 2:** Diet Quality Index-International component scores compared with reference population values.

DQI-I Domain	Reference Value	Mean Score	Reference Population	*p*-Value
Variety	20	13.7 ± 2.8	15.6 ± 0.04	<0.001
Adequacy	40	24.4 ± 5.7	28.1 ± 0.08	<0.001
Moderation	30	17.6 ± 6.1	14.3 ± 0.08	<0.001
Overall balance	10	1.79 ± 2.5	1.1 ± 0.02	0.807
Total score	100	57.6 ± 9.8	59.1 ± 0.14	0.288

Data are presented as the mean ± standard deviation. Statistical analysis was performed using a one-sample *t*-test to compare the observed mean scores with published reference values. A *p*-value < 0.05 was considered statistically significant. Reference population values were those reported by Kim et al. (2003) [2], who originally developed and applied the Diet Quality Index-International (DQI-I).

**Table 3:** Differences according to ASM classification in subjects with MASLD.

Characteristics	Low ASM *n* = 17	Normal ASM *n* = 33	*p*-Value
Age (years)	49 [41–54]	35 [31–45]	0.009
Glucose (mg/dL)	92.5 [84.9–95.0]	93.8 [84.0–105]	0.545
Creatinine (mg/dL)	0.76 [0.63–0.88]	0.85 [0.70–1.03]	0.066
TG (mg/dL)	163 [154–198]	139 [102–179]	0.068
TC (mg/dL)	201 [171–226]	176 [158–197]	0.054
HDL-C (mg/dL)	48.0 [41.6–53.0]	48.0 [39.3–55.8]	0.743
LDL-C (mg/dL)	140 [100–149]	109 [79.0–120]	0.036
TL (mg/dL)	258 [230–331]	260 [156–361]	0.612
AST (U/L)	18 [17–24]	23 [17–40]	0.034
ALT (U/L)	18 [14–29]	30 [20–38]	0.006
GFR (mL/min/1.73 m^2^)	102 [86.5–111]	105 [83.1–118]	0.681
SBP (mm Hg)	123 [115–136]	119 [111–133]	0.760
DBP (mm Hg)	83 [77–86]	81 [75–91]	0.800
Height (cm)	145 [144–151]	161 [154–168]	<0.001
BW (kg)	68.1 [59.7–73.6]	91.6 [83.4–103]	<0.001
BMI (Kg/m^2^)	29.9 [27.1–35.3]	35.1 [33.0–38.5]	0.001
WC (cm)	92.5 [87.0–99.1]	103 [98.5–111]	<0.001
Body fat (%)	46.3 [41.1–51.0]	45.2 [38.7–49.3]	0.566
Fat mass (kg)	31.9 [24.9–37.1]	42.4 [35.5–47.8]	<0.001
Lean mass (kg)	19.3 [17.8–20.5]	30.3 [24.8–32.2]	<0.001
Proteins (g)	50.8 [39.85–59.8]	70.3 [ 56.5–96.0]	0.004
Proteins (%)	13.5 [12.0–15.2]	16.0 [14.0–25.0]	0.009
Lipids (g)	35.9 [22.7–53.8]	56.0 [48.6–67.3]	0.015
Lipids (%)	23.0 [20.0–28.2]	29.0 [27.0–34.0]	0.007
CHO (g)	238 [193–263]	216 [159–269]	0.492
CHO (%)	64.0 [55.3–68.3]	53.0 [45.0–57.1]	<0.001
Energy intake (Kcal/day)	1516 [1249–1722]	1673 [1358–1962]	0.357

Data are presented as the median and interquartile range [Q1–Q3]. Statistical analysis was performed using the Mann–Whitney U test. A *p*-value < 0.05 was considered statistically significant. TG: Triglycerides; TC: Total cholesterol; HDL-C: High-density lipoprotein cholesterol; LDL-C: Low-density lipoprotein cholesterol; TL: total lipids; AST: Aspartate aminotransferase; ALT: Alanine aminotransferase; GFR: Glomerular filtration rate; SBP: Systolic blood pressure; DBP: Diastolic blood pressure; BW: Body weight; BMI: Body mass index; WC: Waist circumference; CHO: Carbohydrates.

**Table 4:** Characteristics of subjects with MASLD according to MetS status.

Characteristics	Non-MetS *n* = 21	MetS *n* = 29	*p*-Value
Age (years)	41 [31–51]	39 [33–49]	0.874
Glucose (mg/dL)	89.6 [82.0–94.0]	98.6 [87.1–107]	0.044
Creatinine (mg/dL)	0.71 [0.65–0.89]	0.88 [0.70–1.08]	0.050
TG (mg/dL)	131 [100–163]	161 [139–198]	0.051
TC (mg/dL)	181 [155–213]	196 [163–210]	0.753
HDL-C (mg/dL)	50.1 [41.7–60.5]	47.0 [36.7–54]	0.156
LDL-C (mg/dL)	102 [79.0–136]	117 [92–144]	0.548
TL (mg/dL)	243 [149–331]	272 [227–361]	0.494
AST (U/L)	21.5 [17.4–25.0]	22.0 [17.4–39.0]	0.745
ALT (U/L)	29.0 [16.0–31.4]	25.0 [17.0–38.5]	0.882
GFR (mL/min/1.73 m^2^)	110.0 [93–118]	99.5 [73.9–118]	0.215
SBP (mm Hg)	117 [110–123]	131 [115–141]	0.010
DBP (mm Hg)	78 [74–83]	87 [81–82]	0.001
Height (cm)	157 [152–165]	154 [149–165]	0.745
BW (kg)	83.4 [71.9–97.4]	81.8 [71.4–92.3]	0.953
BMI (Kg/m^2^)	34.2 [29.9–36.4]	34.5 [30.6–37.3]	0.503
WC (cm)	99.5 [92.1–103]	103 [92.5–110]	0.443
Body fat (%)	45.8 [38.8–50.4]	45.2 [39.1–49.9]	0.898
Fat mass (Kg)	40.8 [30.3–45.4]	37.1 [32.3–44.8]	0.644
Lean mass (Kg)	26.4 [19.7–30.8]	25.0 [20.5–30.9]	0.952
Proteins (g)	60.0 [53.8–75.5]	61 [48.4–91.7]	0.515
Proteins (%)	15 [12–17]	16 [14–22]	0.125
Lipids (g)	52.8 [36.2–65.5]	52.2 [36.8–67.5]	0.864
Lipids (%)	29 [26–33]	28 [24–40]	0.967
CHO (g)	254 [214–284]	198 [158–246]	0.034
CHO (%)	57 [54–64]	53 [44–63]	0.079
Energy intake (Kcal/day)	1638 [1411–1990]	1518 [1238–1793]	0.297

Data are presented as the median and interquartile range [Q1–Q3]. Statistical analysis was performed using the Mann–Whitney U test. A *p*-value < 0.05 was considered statistically significant. TG: Triglycerides; TC: Total cholesterol; HDL-C: High-density lipoprotein cholesterol; LDL-C: Low-density lipoprotein cholesterol; TL: Total lipids; AST: Aspartate aminotransferase; ALT: Alanine aminotransferase; GFR: Glomerular filtration rate; SBP: Systolic blood pressure; DBP: Diastolic blood pressure; BW: Body weight; BMI: Body mass index; WC: Waist circumference; CHO: Carbohydrates.

**Table 5:** Comparison of metabolic, anthropometric, body composition, and dietary characteristics according to the combined metabolic syndrome and appendicular skeletal muscle mass classification in participants with MASLD.

Characteristics	Non-MetS *n* = 21		MetS *n* = 29		*p*-Value
	Low ASM *n* = 7	Normal ASM *n* = 14	Low ASM *n* = 10	Normal ASM *n* = 19	
Age (years)	49 [41–52]	33 [31–44]	48 [39–58]	35 [32–44]	0.066
Glucose (mg/dL)	92.5 [83.0–94.1]	89.3 [82.3–93.9]	93.1 [89.7–99.4]	100 [86.5–110]	0.171
Creatinine (mg/dL)	0.65 [0.56–0.79]	0.7 [0.7–0.9]	0.80 [0.68–0.97]	0.96 [0.75–1.14]	0.058
TG (mg/dL)	163 [153–203] ^a^	104 [88.1–136] ^b^	162 [155–194] ^a^	161 [135–201] ^a^	0.018
TC (mg/dL)	182 [174–225]	178 [134–209]	212 [179–226]	173 [163–197]	0.228
HDL-C (mg/dL)	50.1 [44.8–57.1]	49.9 [41.3–59.1]	46.5 [42.0–49.8]	47.7 [36.3–54.9]	0.539
LDL-C (mg/dL)	125 [98.1–141]	94.4 [77.9–130]	146 [111–149]	109 [82.5–120]	0.157
TL (mg/dL)	278 [233–333]	207 [127–321]	248 [231–297]	277 [221–365]	0.493
AST (U/L)	17.4 [16.5–24.5]	22.3 [18.3–26.3]	18.0 [17.5–21.4]	25.0 [17.5–48.6]	0.188
ALT (U/L)	28.0 [14.5–29.5] ^ab^	30.3 [21.8–34.4] ^a^	17.9 [11.4–24.0] ^b^	32.0 [18.7–45.0] ^a^	0.040
GFR (mL/min/1.73 m^2^)	110 [97.9–112]	112 [92.9–118]	94.0 [80.4–111]	104.0 [69.2–119]	0.631
SBP (mm Hg)	120 [110–123]	112 [110–122]	130 [119–140]	130 [113–141]	0.078
DBP (mm Hg)	76.0 [74.0–83.3] ^b^	79.5 [74.0–80.5] ^b^	85.5 [82.3–91.6] ^a^	88.5 [79.5–92.0] ^ab^	0.015
Height (cm)	145 [144–149] ^b^	164 [157–169] ^a^	146 [144–152] ^b^	161 [154–167] ^a^	<0.001
BW (kg)	64.8 [59.8–72.2] ^b^	91.5 [83.6–105] ^a^	69.5 [60.2–72.9] ^b^	91.6 [85.9–99.3] ^a^	<0.001
BMI (Kg/m^2^)	29.9 [27.1–32.9] ^b^	35.1 [31.7–37.3] ^ab^	29.6 [27.6–34.3] ^b^	35.9 [33.3–39.0] ^a^	0.011
WC (cm)	91.0 [85.5–99] ^b^	102 [98.6–107] ^a^	92.6 [89.5–98.3] ^b^	107 [101–111] ^a^	0.001
Body fat (%)	46.3 [42.8–49.4]	44.6 [38.3–49.6]	42.85 [41.4 –50.8]	45.8 [38.9–49.3]	0.906
Fat mass (Kg)	31.9 [28.1–36.5] ^b^	43.2 [37.2–48.0] ^a^	31.2 [24.6–36.9] ^b^	40.2 [35.4–46.9] ^a^	0.008
Lean mass (Kg)	19.3 [17.1–19.7] ^b^	30.3 [27.0–32.4] ^a^	19.4 [18.6–20.5] ^b^	28.9 [24.5–31.9] ^a^	<0.001
Proteins (g)	47.9 [38.9–58.3] ^b^	66.7 [55.3–75.9] ^a^	50.8 [41.8–60.4] ^b^	77.0 [59.35–107] ^a^	0.026
Proteins (%)	12.0 [11.3–13.5] ^b^	15.0 [13.5–17.8] ^ab^	14.5 [13.0–15.7] ^b^	20.0 [14.0–26.5] ^a^	0.023
Lipids (g)	35.9 [32.7–39.6]	55.9 [49.8–65.7]	35.6 [20.3–57.2]	56.0 [44.9–72.3]	0.105
Lipids (%)	22.5 [20.5–25.3] ^b^	30.5 [28.3–33.8] ^a^	24.5 [20.8–34.8] ^ab^	28.0 [26.0–39.8] ^a^	0.029
CHO (g)	278 [234–284]	241 [198–281]	226 [187–244]	178 [155–266]	0.138
CHO (%)	67.5 [63.2–68.8] ^a^	55.5 [51.8–60.0] ^b^	62.0 [53.8–65.8] ^ab^	46.0 [40.5–54.0] ^b^	0.001
Energy intake (Kcal/day)	1636 [1381–1802]	1652 [1431–2017]	1397 [1166–1674]	1673 [1255–1795]	0.561

Data are presented as the median and interquartile range [Q1–Q3]. Comparisons among the four groups were performed using the Kruskal–Wallis test followed by Dunn’s post hoc test. A *p* < 0.05 was considered statistically significant. Different superscript letters indicate statistically significant differences between groups; groups sharing at least one superscript letter were not significantly different, where a > b > c. TG: Triglycerides; TC: Total cholesterol; HDL-C: High-density lipoprotein cholesterol; LDL-C: Low-density lipoprotein cholesterol; TL: Total lipids; AST: Aspartate aminotransferase; ALT: Alanine aminotransferase; GFR: Glomerular filtration ratio; SBP: Systolic blood pressure; DBP: Diastolic blood pressure; BW: Body weight; BMI: Body mass index; WC: Waist circumference; CHO: Carbohydrates.

## Data Availability

The original contributions presented in this study are included in the article and Supplementary Materials. Further inquiries can be directed to the corresponding author.

## Supplementary Materials

The following supporting information can be downloaded at: https://www.mdpi.com/article/10.3390/metabo16080546/s1, Supplementary Table S1: Normality test of the study variables; Supplementary Table S2: Differences according to ASM/height^2^ classification in subjects with MASLD; Supplementary Table S3: Exploratory ASM/height2 threshold for discriminating metabolic syndrome based on ROC analysis; Supplementary Table S4: Associations between DQI-I domains and ASM classification in subjects with MASLD; Supplementary Figure S1: Exploratory ROC curve of ASM/height^2^ for discriminating metabolic syndrome.

## Abbreviations

The following abbreviations are used in this manuscript:

MASLD	Metabolic dysfunction-associated steatotic liver disease
ASM	Appendicular skeletal muscle mass
MetS	Metabolic syndrome
DQI-I	Diet Quality Index-International
ALM	Appendicular lean mass
NAFLD	Non-alcoholic fatty liver disease
EWGSOP2	European Working Group on Sarcopenia in Older People
T2D	Type 2 diabetes
HT	Hypertension
UL	Urolithiasis
GFR	Glomerular filtration rate
SBP	Systolic blood pressure
DBP	Diastolic blood pressure
TG	Triglycerides
TC	Total cholesterol
HDL-C	High-density lipoprotein cholesterol
LDL-C	Low-density lipoprotein cholesterol
TL	Total lipids
AST	Aspartate aminotransferase
ALT	Alanine aminotransferase
BW	Body weight
BMI	Body mass index
WC	Waist circumference
CHO	Carbohydrates
