# Supplementary material for: The Relationships Between Diet Quality Index, Appendicular Skeletal Muscle Mass, and Metabolic Syndrome in a Population with Metabolic Dysfunction-Associated Steatotic Liver Disease: A Single Center from the Yucatan Peninsula
Source: Metabolites. 2026 Aug 1;16(8):546. doi: 10.3390/metabo16080546 (PMC13515144; doi:10.3390/metabo16080546)
Supplement: Supplementary file 1 [file metabolites-16-00546-s001.zip › Supplementary Tables.pdf]

Supplementary Table S1. Normality test of the study variables.

Characteristics	Shapiro-Wilk W	*p*-value	Distribution
Age (years)	0.938	0.011	Non-normal
Glucose (mg/dL)	0.896	< 0.001	Non-normal
Creatinine (mg/dL)	0.882	< 0.001	Non-normal
TG (mg/dL)	0.808	< 0.001	Non-normal
TC (mg/dL)	0.967	0.017	Non-normal
HDL-C (mg/dL)	0.970	0.053	Normal
LDL-C (mg/dL)	0.966	0.016	Non-normal
TL (mg/dL)	0.884	< 0.001	Non-normal
AST (U/L)	0.670	< 0.001	Non-normal
ALT (U/L)	0.437	< 0.001	Non-normal
GFR (mL/min/1.73 m^2)	0.903	< 0.001	Non-normal
SBP (mm Hg)	0.940	0.015	Non-normal
DBP (mm Hg)	0.986	0.049	Non-normal
Height (cm)	0.955	0.048	Non-normal
BW (kg)	0.974	0.132	Normal
BMI (Kg/m^2)	0.962	0.011	Non-normal
WC (cm)	0.936	0.009	Non-normal
Body fat (%)	0.954	0.048	Non-normal
Fat mass (Kg)	0.976	0.040	Non-normal
Lean mass (Kg)	0.959	0.370	Normal
Proteins (g)	0.863	< 0.001	Non-normal
Proteins (%)	0.823	< 0.001	Non-normal
Lipids (g)	0.854	0.010	Non-normal
Lipids (%)	0.872	< 0.001	Non-normal
CHO (g)	0.863	< 0.001	Non-normal
CHO (%)	0.964	< 0.001	Non-normal
Energy intake (Kcal/day)	0.986	0.819	Normal

Normality was assessed using the Shapiro–Wilk test. Variables with $p < 0.05$ were considered to deviate from a normal distribution. For consistency, all continuous variables are presented as median and interquartile range [Q1–Q3] in the manuscript, regardless of their distribution. TG: Triglycerides; TC: Total cholesterol; HDL-C: High-density lipoproteins cholesterol; LDL-C: Low-density lipoproteins cholesterol; TL: total lipids; AST: Aspartate aminotransferase; ALT: Alanine aminotransferase; GFR: Glomerular filtration rate; SBP: Systolic blood pressure; DBP: Diastolic blood pressure; BW: Body weight; BMI: Body mass index; WC: Waist circumference; CHO: Carbohydrates.

Supplementary Table S2. Differences according to ASM/height^2 classification in subjects with MASLD.

Characteristics	Low ASM/height^2 n = 2	Normal ASMheight^2 n = 48	p-value
Age (years)	54 [47–60]	39 [31–49]	0.197
Glucose (mg/dL)	86.8 [83.9–89.7]	93.5 [84.2 –103]	0.413
Creatinine (mg/dL)	0.52 [0.50–0.52]	0.80 [0.70–1.01]	0.019
TG (mg/dL)	145 [125–165]	156 [114–183]	0.940
TC (mg/dL)	156 [135–178]	186 [162–214]	0.442
HDL-C (mg/dL)	47.3 [44.5–50.7]	48.0 [39.9–56.3]	1.000
LDL-C (mg/dL)	92.9 [74.4–111]	109 [82.8–145]	0.442
TL (mg/dL)	306 [292–321]	250 [200–338]	0.471
AST (U/L)	16.7 [16.4–17.1]	22.1 [17.6–29.5]	0.137
ALT (U/L)	12.8 [12.2–13.4]	28.5 [17.3–35.3]	0.066
GFR (mL/min/1.73 m^2)	110 [106–115]	105 [84.1–118]	0.503
SBP (mm Hg)	124 [124–125]	120 [111–137]	0.667
DBP (mm Hg)	74.3 [68.6–79.9]	82.5 [76.5–90.8]	0.404
Height (cm)	143 [142–143]	157 [151–165]	0.024
BW (kg)	53.5[52.3–54.6]	83.7 [73.2–93.7]	0.001
BMI (Kg/m^2)	26.2 [25.7–26.6]	34.8 [30.6–37.2]	0.031
WC (cm)	80.9 [79.4–82.5]	101 [92.6–107]	0.024
Body fat (%)	42.8 [40.9–44.5]	45.5 [38.8–50.4]	0.747
Fat mass (kg)	22.9 [21.5–24.5]	38.5 [32.2–45.4]	0.044
Lean mass (kg)	16.05 [15.9–16.2]	25.9 [21.1–30.9]	0.018
Proteins (g)	115 [115–116]	60.9 [49.6–84.5]	N/A
Proteins (%)	21.0 [21.0–21.0]	15.0 [13.1–20.2]	N/A
Lipids (g)	70.1 [70.1–70.1]	52.2 [36.2–71.1]	N/A
Lipids (%)	29.0 [29.0–29.0]	28.0 [24.0–34.3]	N/A
CHO (g)	283 [283–283]	224 [163–268]	N/A
CHO (%)	52.0 [52.0–52.0]	55.0 [45.8–64.0]	N/A
Energy intake (Kcal/day)	2186 [2186–2186]	1591 [1266–1843]	N/A

Data are presented as median and interquartile range [Q1–Q3]. Statistical analysis was performed using the Mann–Whitney U test. A p-value < 0.05 was considered statistically significant. TG: Triglycerides; TC: Total cholesterol; HDL-C: High density lipoproteins cholesterol; LDL-C: Low density lipoproteins cholesterol; TL: total lipids; AST: Aspartate aminotransferase; ALT: Alanine aminotransferase; GFR: Glomerular rate ratio; SBP: Systolic blood pressure; DBP: Diastolic blood pressure; BW: Body weight; BMI: Body mass index; WC: Waist circumference; CHO: Carbohydrates; N/A: Not applicable.

Supplementary Table S3. Exploratory ASM/height^2 threshold for discriminating metabolic syndrome based on ROC analysis.

Comparison	Exploratory ASM/height2 threshold	Sensitivity (%)	Specificity (%)	Youden Index	AUC	95% CI
MetS vs non-MetS	7.56	50.0	80.9	0.31	0.679	0.52–0.82

Exploratory ROC analysis using metabolic syndrome (MetS) as the reference outcome. The optimal threshold was determined according to Youden's index. Because this analysis was exploratory and based on a single-center cohort, the proposed threshold should not be interpreted as a definitive diagnostic cut-off and requires external validation.

Supplementary Table S4. Associations between DQI-I domains and ASM classification in subjects with MASLD.

DQI-I domain	Low ASM *n* = 16	Normal ASM *n* = 33	*p*-value
Variety	13.0 [12.0–15.5]	14.0 [12.0–16.1]	0.566
Adequacy	25.6 [21.9–31.6]	24.6 [21.8–27.8]	0.492
Moderation	21.0 [20.2–24.1]	15.0 [12.0–21.2]	0.019
Overall balance	2.0 [0.0–4.0]	0.0 [0.0–2.0]	0.080
Total score	62.3 [55.3–68.5]	57.0 [52.0–62.2]	0.040

Data are presented as median and interquartile range [Q1–Q3].
